# Embryonic diapause due to high glucose is related to changes in glycolysis and oxidative phosphorylation, as well as abnormalities in the TCA cycle and amino acid metabolism

**DOI:** 10.3389/fendo.2023.1135837

**Published:** 2023-12-18

**Authors:** Jiewei Hong, Hongxuan Tong, Xuan Wang, Xiaoyan Lv, Lijuan He, Xuezhi Yang, Yingli Wang, Kaixia Xu, Qi Liang, Qianjin Feng, Tingli Niu, Xin Niu, Yu Lu

**Affiliations:** ^1^ School of Traditional Chinese Medicine, Beijing University of Chinese Medicine, Beijing, China; ^2^ Institute of Basic Theory of Chinese Medicine, China Academy of Chinese Medical Sciences, Beijing, China; ^3^ Party Committee Office, Shanxi Health Vocational College, Shanxi, China; ^4^ Library Collection and Editing Department, Beijing University of Chinese Medicine, Beijing, China; ^5^ Rehabilitation Department, Dongfang Hospital Beijing University of Chinese Medicine, Beijing, China; ^6^ Experimental Management Center, Shanxi University of Traditional Chinese Medicine, Shanxi, China; ^7^ School of Basic Medicine, Shanxi University of Traditional Chinese Medicine, Shanxi, China; ^8^ Centre for Marine Bioproducts Development, College of Medicine and Public Health, Flinders University, Bedford Park, SA, Australia; ^9^ Medical Insurance Office, Beijing University of Chinese Medicine Third Affiliated Hospital, Beijing, China; ^10^ Institute of Information on Traditional Chinese Medicine, China Academy of Chinese Medical Sciences, Beijing, China

**Keywords:** embryo arrest, high glucose, embryo metabolism, *in vitro*, GLUTs, GC-MS

## Abstract

**Introduction:**

The adverse effects of high glucose on embryos can be traced to the preimplantation stage. This study aimed to observe the effect of high glucose on early-stage embryos.

**Methods and results:**

Seven-week-old ICR female mice were superovulated and mated, and the zygotes were collected. The zygotes were randomly cultured in 5 different glucose concentrations (control, 20mM, 40mM, 60mM and 80mM glucose). The cleavage rate, blastocyst rate and total cell number of blastocyst were used to assess the embryo quality. 40 mM glucose was selected to model high glucose levels in this study. 40mM glucose arrested early embryonic development, and the blastocyst rate and total cell number of the blastocyst decreased significantly as glucose concentration was increased. The reduction in the total cell number of blastocysts in the high glucose group was attributed to decreased proliferation and increased cell apoptosis, which is associated with the diminished expression of GLUTs (GLUT1, GLUT2, GLUT3). Furthermore, the metabolic characterization of blastocyst culture was observed in the high-glucose environment.

**Discussion:**

The balance of glycolysis and oxidative phosphorylation at the blastocyst stage was disrupted. And embryo development arrest due to high glucose is associated with changes in glycolysis and oxidative phosphorylation, as well as abnormalities in the TCA cycle and amino acid metabolism.

## Introduction

Spontaneous abortion and congenital malformations are associated with high maternal glucose (1). Clinical observations strongly support the link between fetal loss, perinatal deaths and congenital birth defects and poor glycemic control (2, 3). Evidence has shown that elevated blood glucose and glycosylated hemoglobin levels during early pregnancy in diabetic mothers lead to an increased risk of fetal malformations and spontaneous abortion (4–6). What’s worse, such adverse effects caused by high glucose on the offspring of diabetic mothers can be traced to the early embryonic development stages (7). Apoptosis-controlling genes are activated at the blastocyst stage when subjected to hyperglycemic conditions (8). In addition, high glucose-induced abnormal embryonic development occurs even in the absence of systemic high maternal glucose (9). Exposure to a hyperglycemic environment for 24 h was sufficient to cause spontaneous abortion and congenital malformations (10, 11).

The sensitivity of embryos to blood glucose fluctuation is not yet fully understood. Study of abnormal embryonic development is usually based on rodent models. Most of the existing studies mainly focus on the following aspects: mitochondrial dysfunction (12–14), oxidative stress (15–24), excessive cell death or apoptosis related to glucose transporters (8, 23, 25–36), autophagy (37, 38), epigenetic mechanisms (39–45) and gene expression (46, 47). Among them, mitochondrial dysfunction, oxidative stress and apoptotic cell death (event 1-3) might represent different aspects of a dimension. The preimplantation embryo stage or early pregnancy stage may be critical for diabetic teratogenesis, which has not been adequately explored in the *in vitro* rodent model. Moreover, few studies have explored metabolic alterations of those embryos coping with the high glucose environment.

Metabonomics has been increasingly applied as an adjunct technique to morphology. This non-invasive method is widely used to evaluate the quality of preimplantation embryos and has achieved good outcomes (48–52). Embryo viability and embryo developmental potential can be predicted by amino acid analysis in the embryo culture medium (53, 54). Furthermore, metabolic alterations in high maternal glucose can deepen our understanding of high glucose-induced abnormal embryonic development. Cause phenotypic and metabolic abnormalities have been found in embryos from overweight and obese women (55). Therefore, applying this technology to study metabolic alterations in a diabetic-like environment may provide further insight into abnormal embryonic development arrest due to high glucose.

## Materials and methods

### Ethical approval

All experiments were approved by the Beijing University of Chinese Medicine Animal Ethics Committee and conducted in accordance with the Code of Practice for the Care and Use of Animals for Scientific Purposes.

### Experimental design

(1) The embryotoxic and teratogenic effects of glucose on embryonic development were investigated with different concentrations of glucose. (2) Using embryo quality assessment, an appropriate concentration of glucose was selected in subsequent experiments. (3) The culture medium at the blastocyst stage was collected and assessed by metabolomic analysis.

### Animal handling and embryo culture

ICR mice were housed in a pathogen-free room at the Laboratory Animal Center of Beijing University of Chinese Medicine. They were given access to food and water ad libitum and maintained in 12 h light/dark cycle with standard temperature (22 ± 1°C) and humidity (55 ± 10%). After one week of acclimatization, 7-week-old mice were superovulated by intraperitoneal injections of 7.5 IU of equine chorionic gonadotropin (eCG, Ningbo Hormone Products Co.), followed by 7.5 IU of human chorionic gonadotropin (hCG; Ningbo Hormone Products Co.) 48 hours later. They were boxed individually with stud males of the same strain with proven fertility for mating. 10h after hCG injection, the presence of copulation plugs was confirmed in the female mice. Fourteen hours after the hCG injection, the females with copulation plugs were culled via cervical dislocation. The oviductal ampulla was removed and placed in an M2 medium supplemented with 4 mg/mL bovine serum albumin (BSA). The enlarged fallopian tube kept at a heating plate of 37°C was torn with a sterile needle under a stereomicroscope (Olympus SZ61). Subsequently, cumulus cells were removed with hyaluronidase (300 μg/mL, 3 minutes digestion), and zygotes were collected using a mouth-controlled micropipette. After cleaning, those zygotes were randomly allocated to droplets of KSOM medium, which were covered with mineral oil and equilibrated overnight prior to use. The zygotes were randomly assigned to five groups: (1) control containing 2.81 mM glucose; (2) 20 mM glucose; (3) 40 mM glucose; (4) 60 mM glucose; (5) 80 mM glucose. Around 20 embryos were placed in each 75μL drop of culture and cultured in a CO^2^ incubator at 37°C and 5% CO^2^ saturation humidity for 5 days (Thermo 311). The manipulations were performed very carefully on a heated microscope stage (37°C) and at room temperature (20-25°C) in order to minimize environmental stimulus. 140-160 embryos were collected from 8 selected female mice for each experiment, and all experiments were repeated four times. All the chemicals used for the medium system were obtained from Sigma Co. (St. Louis, MO, USA) unless indicated. The formulations are detailed in **Supplementary Tables 1**, **2**.

### Embryo quality assessment

Embryo quality was assessed by morphology, cleavage rate, blastocyst rate, and the total cell number of blastocysts. 2-cell embryos were observed at 42h post-hCG injection, while blastocysts were observed at 114h post-hCG injection (14:00 on Day 5 and 14:00 on Day 8). Cleavage rate = number of 2-cell embryos/total number of zygotes; Blastocyst rate = number of blastocysts/number of 2-cell embryos. The cleavage rate was significantly affected by fertilization status, while the blastocyst rate was relatively unaffected. To determine the total cell number of the blastocysts, the blastocysts were fixed with 4% paraformaldehyde for 30 min at 4°C, washed three times with PBS and stained blue with 0.1 µg/ml 4’,6-diamidino-2-phenylindole dihydrochloride (DAPI; Boehringer, Mannheim, Germany) for 10 min. The cells were arranged on a glass slide, covered with a coverslip, and sealed with nail polish. The stained blastocysts were photographed with an Olympus BX60 microscope and analyzed using ImageJ software. Nuclei were counted three times per blastocyst by two different investigators blinded to the different groups. The nuclei counts were the same between the two investigators, substantiating the reliability of this method. The detection of cell proliferation and apoptosis was performed according to an Edu staining kit (Beyotime). And the dosage of EDU labeling was 200mg/kg, intraperitoneally injected 4 hours before mice were culled (n=20-40 embryos per treatment).

### Quantitative analysis of GLUTs protein using western blot

Collected blastocysts in groups of 20-40 for protein extraction following the Protein Extraction Kit instructions (GenePool/GPP1815). Adjusted the protein concentration and denatured by boiling at 100 °C for 10 minutes. Prepared 12% separation gel and 5% concentration gel based on the target protein’s molecular weight following the SDS-PAGE Gel Kit instructions (GenePool/GPP1816). Performed immunoblotting experiments, including transferring to a PVDF membrane, immersing in Milk Blocking Buffer (GenePool/GPP1819)/BSA Blocking Buffer (GenePool/GPP1818), and incubating with diluted primary antibodies (GLUT1 (Beyotime, AF1015, 1:500), GLUT2 (Beyotime, AG3238, 1:500), GLUT3 Polyclonal antibody (Proteintech, 20403-1-AP, 1:1000). Diluted the secondary antibody with Milk Blocking Buffer (GenePool/GPP1819), sheep anti-rabbit HRP (based on the source of the primary antibody) at a 1:5000 dilution and incubated at room temperature for 50 minutes. Immersed the PVDF film in the ECL (GenePool/GPP1824) color solution for 1 minute, followed by exposure, development, and fixation in a dark room.

### Sample collection and preparation for GC/MS analysis

Blastocysts were collected, and all the embryos were removed from the culture at 114h post-hCG injection using a mouth-controlled micropipette. The culture droplets were snap-frozen in liquid nitrogen and stored at -80°C. 40 mM glucose was reliable and effective as a half-inhibitory dose to inhibit embryonic development. Therefore, the culture medium of the control groups and the 40 mM glucose groups were selected for gas chromatography (GC) and mass spectrometry (MS) analyses. First, 100μL of culture medium was mixed with 0.35ml methanol and 20μL of L-2-chlorophenylalanine (1mg/mL stock in dH2O) as internal standard and centrifuged for 15min at 13000rpm, 4°C. Then, 0.4ml of the supernatant was carefully transferred into a fresh 2ml GC/MS glass vial, and 11μL of each sample was taken and pooled as a QC sample. All the samples were vacuum-dried without heating. Then, 60μL of methoxy amination hydrochloride (20mg/mL in pyridine) was added, and the aliquots were incubated for 30min at 80 °C. 80μL of the BSTFA regent (1% TMCS, v/v) was added, and the aliquots were incubated for 2h at 70 °C. Finally, 10μL FAMEs (standard mixture of fatty acid methyl esters, C8-C16:1mg/mL, C18-C24:0.5mg/mL in chloroform) was added, and the derivatized samples were cooled to room temperature prior to GC-MS analysis.

### GC-MS analysis

GC/TOF-MS analysis was performed using an Agilent 7890A gas chromatograph system (Agilent 7890A, Agilent, USA) coupled with a Pegasus 4D time-of-flight mass spectrometer (LECO Chroma TOF PEGASUS 4D, LECO, USA). The system utilized a DB-5MS capillary column coated with 5% diphenyl cross-linked with 95% dimethylpolysiloxane (30m×250μm inner diameter, 0.25μm film thickness; J&W Scientific, Folsom, CA, USA). A 1μL aliquot of the analyte was injected in splitless mode. Helium was used as the carrier gas, the front inlet purge flow was set to 3mL/min, and the gas flow rate through the column was set to 1mL/min. The initial temperature was kept at 80°C for 1 min, then raised to 290°C at a rate of 10°C/min, then kept for 12min at 290°C. The injection, transfer line, and ion source temperatures were 280, 270, and 220°C, respectively. The energy was set to -70eV in electron impact mode. Finally, the mass spectrometry data were acquired in full-scan mode with the m/z range of 50-600 at a rate of 12spectra per second after a solvent delay of 468s.

### Multivariate statistical analysis, metabolite identification and pathway analysis

The analyses were performed according to the previous research (56). First, peaks were detected, and metabolites were left through the interquartile range denoising method. The missing values in the raw data were filled up by half of the minimum value, and the internal standard normalization method was employed. Then, the resulting three-dimensional data involving the peak number, sample name, and normalized peak area were fed to the SIMCA14.0 software package (MKS Data Analytics Solutions, Umea, Sweden). Principal component analysis (PCA) was performed for the unsupervised analysis. Supervised orthogonal projections to latent structures-discriminate analysis (OPLS-DA) were applied to obtain a higher level of group separation and a better understanding of variables responsible for classification. R^2^X: variation determined by the OPLS-DA model; R^2^Y: validity of the model; Q2: predictive accuracy of the model. Subsequently, the differential metabolites between the control and glucose groups were distinguished (VIP > 1 and P <0.05). The greater the difference in metabolite levels between groups, the more important that metabolite become in creating the final model, as reflected by the VIP value. VIP values exceeding 1.0 were first selected as significant metabolites. Those selected variables were assessed by Student’s t-test, with P values <0.05 considered to be the differential metabolites. After that, the enrichment and topology analysis were performed. Commercial databases, including KEGG (http://www.genome.jp/kegg/) and NIST (http://www.nist.gov/index.html) were utilized to search for the pathways based on those differential metabolites.

### Data processing and analysis

All analyses were performed using GraphPad Prism 9.01 (GraphPad Software Inc, San Diego, CA, USA). Data were represented as (1) median, maximum and minimum; (2) mean ± SEM, with p < 0.05 considered statistically significant. Statistical analysis of significant differences was performed using student’s t-test, ANOVA or non-parametric test where appropriate.

## Results

### Morphological events and cleavage rate of embryos cultured in high glucose

**Figure 1:**
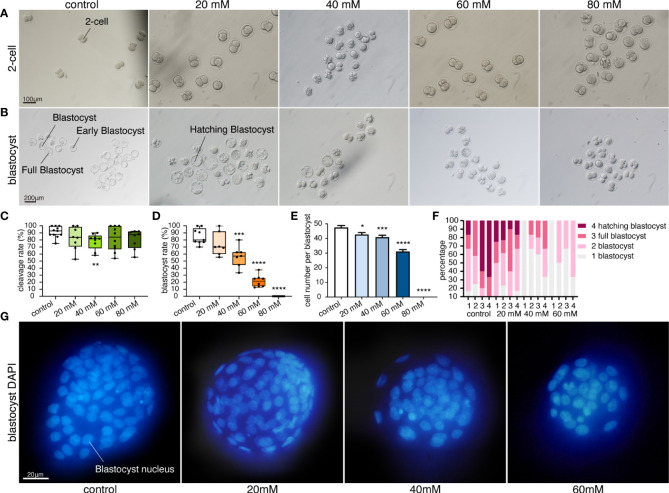
High glucose concentration inhibits embryonic development. **(A)** Morphology of 2-cell embryos cultured in five glucose concentrations. Scale bar, 100μm. **(B)** Morphology of blastocysts cultured in five glucose conditions. Scale bar, 200μm. **(C)** Cleavage rates in different glucose concentrations. Each dot represents a calculated cleavage rate (cleavage rate derived from at least three treatments per experiment and three independent experiments, n= at least 10 embryos per treatment). **p=0.0053. **(D)** Blastocyst rates in different glucose concentrations. Box-and-whiskers plots showing the median, interquartile ranges (box) and minimum and maximum ranges (whiskers). Each dot represents a blastocyst rate (n = at least 10 embryos per treatment, blastocyst rate derived from at least three treatments per experiment and three independent experiments. **p=0.0006, ****p<0.0001. **(E)** Total cell number per blastocyst cultured under four glucose conditions. Values are expressed as mean ± SEM. n= at least 20 blastocysts per treatment from four independent biological replicates. *p=0.0125, ***p=0.0005, ****p<0.0001. **(F)** Stacked bar plots showing the percentage of blastocysts in that stage under different glucose conditions. A stacked bar of different colors represents the proportion of the blastocyst in that blastocyst stage. The proportion of the blastocyst was calculated by repeating the measurements 4 times per group (n = at least 10 embryos per treatment). 4 different stage blastocysts were calculated in our experiment. Developmental stages: 1 Early blastocyst; the blastocoel filled more than half the volume of the conceptus, but no expansion in overall size as compared to earlier stages. 2 Blastocyst; the blastocoel filled more than half of the volume of the conceptus; with slight expansion in overall size and notable thinning of the zona pellucida. 3 Full blastocyst; a blastocoel of more than 50% of the conceptus volume and overall size fully enlarged with a very thin zona pellucida. 4 Hatching blastocyst; non-preimplantation genetic diagnosis. The trophectoderm herniating through the zona. 5 Fully hatched blastocyst; non-preimplantation genetic diagnosis. Free blastocyst fully removed from zona pellucida. 6 Hatching or hatched blastocyst; preimplantation genetic diagnosis (Veeck & Zaninovic, 2003 (57)). **(G)** Fluorescence pattern of blastocysts cultured in four glucose concentrations, stained with DAPI. Scale bar, 20μm.

All the 2-cell embryos displayed normal morphology with even blastomere and complete zona pellucida (**Figure 1A**). Cleavage rate was significantly lower in the 40 mM glucose compared to the control group (p=0.0053). However, there was no significant change in the higher concentrations of 60mM or 80mM groups. Thus, exposure to high glucose concentrations had no significant effect on the cleavage rate (**Figure 1C**).

### Morphological events and blastocyst rates of embryos cultured in high glucose

Blastocysts in 20mM glucose showed no significant difference in blastocyst rate compared with the control group (**Figure 1D**). The blastocysts looked complete and full. The blastocyst cavity was fluid-filled, the inner cell mass was tightly packed, and the zona pellucida became thinner due to the expansion. Furthermore, the trophectoderm was clearly distinguishable (**Figure 1B**). The blastocyst rate was significantly decreased in 40mM, 60mM and 80mM glucose. High glucose concentration (more than 40mM glucose) inhibited embryonic development, and a direct correlation between increased glucose concentration and decreased blastocyst rate was observed (**Figure 1D**). Most blastocysts were early blastocysts with small blastocyst cavities and less cavity fluid (**Figure 1B**). In addition, a higher proportion of early low-quality blastocysts was observed in high glucose concentration, with a lower proportion of normal blastocysts. Hatching blastocysts were only seen in the control and 20 mM glucose groups (**Figure 1F**). No hatching blastocyst was observed in glucose concentrations higher than 40mM, and the trophectoderm was indistinguishable. More importantly, no blastocysts were found in 80mM glucose, and the largest number of bad embryos stopped developing at the 2-cell stage (**Figure 1B**).

### Effect of high glucose on the total cell number of blastocysts

Blastocyst nuclei were stained blue (**Figure 1G**), and a significant decrease in total cell number (TCN) was observed in glucose concentrations of more than 20mM. TCN decreased significantly as the glucose concentration in the culture medium was increased (**Figure 1E**). TCN in 20mM glucose decreased (42.68, p=0.0125, **Figure 1E**), but the blastocyst rate showed no significant difference compared to the control group (**Figure 1D**). Moreover, the blastocysts in 60mM glucose were poorly developed with low TCN (**Figure 1E**), and no blastocysts were found in the 80mM glucose groups (**Figure 1D**).

### Embryo arrest and glucose transporters

**Figure 2:**
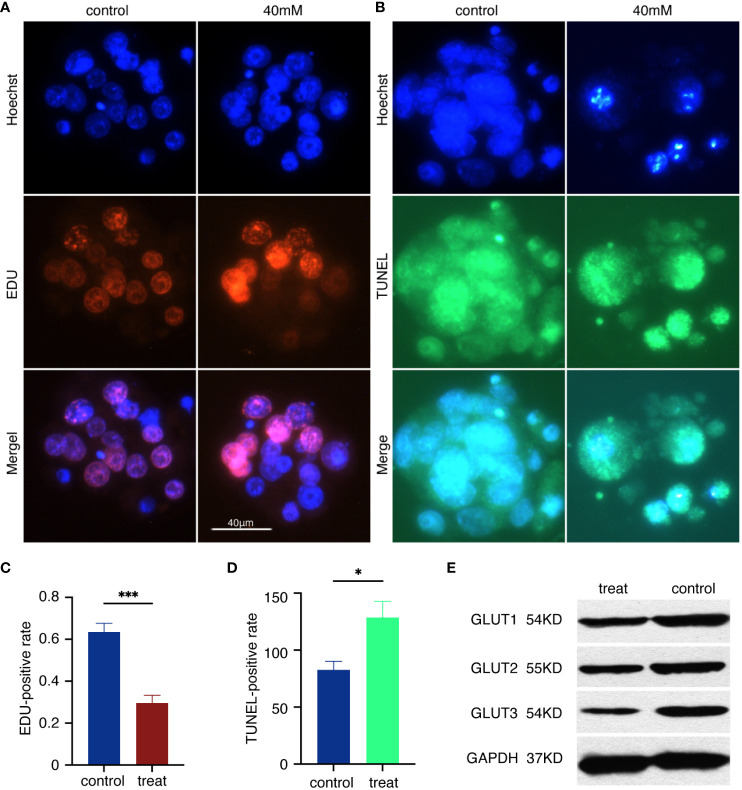
Embryo arrest and glucose transporters. **(A)** Hoechst 33342 staining and EDU staining of the blastocyst. Glowing red blastocyst shows proliferating cells. Scale bar, 40μm. **(B)** Hoechst 33342 staining and TUNEL staining. Glowing green blastocyst shows apoptotic cells. **(C)** Relative EDU positive intensity = the mean EDU intensity of each blastocyst/mean Hoechst intensity of each blastocyst. ***p<0.0001. **(D)** Relative TUNEL positive intensity = mean TUNEL intensity of each blastocyst/mean Hoechst intensity of each blastocyst. *p<0.0217. **(E)**The immune complexes were analyzed by immunoblotting using antibodies against GLUT1, GLUT2 and GLUT3. The protein inputs were detected with western blotting using indicated antibodies.

To assess the level of arrest in the high glucose embryos, the EDU assay and TUNEL assay were performed on normal blastocysts as well as the blastocysts in 40mM glucose. The cell nuclei were stained by Heochst 33342. Compared with the control group, the proportion of EDU-positive markers in the blastocyst nucleus of the 40mM high glucose group decreased (**Figure 2A**), and the relative red fluorescence intensity decreased (**Figure 2C**) The blastocysts in the high glucose group showed more abundant TUNEL positive signals (**Figure 2B**) and increased relative green fluorescence intensity (**Figure 2D**). GLUT1, GLUT2 and GLUT3 of the blastocyst inputs were detected with western blotting using indicated antibodies (**Figure 2E**).

### Multivariate data analysis of metabolites in culture medium based on GC-MS

**Figure 3:**
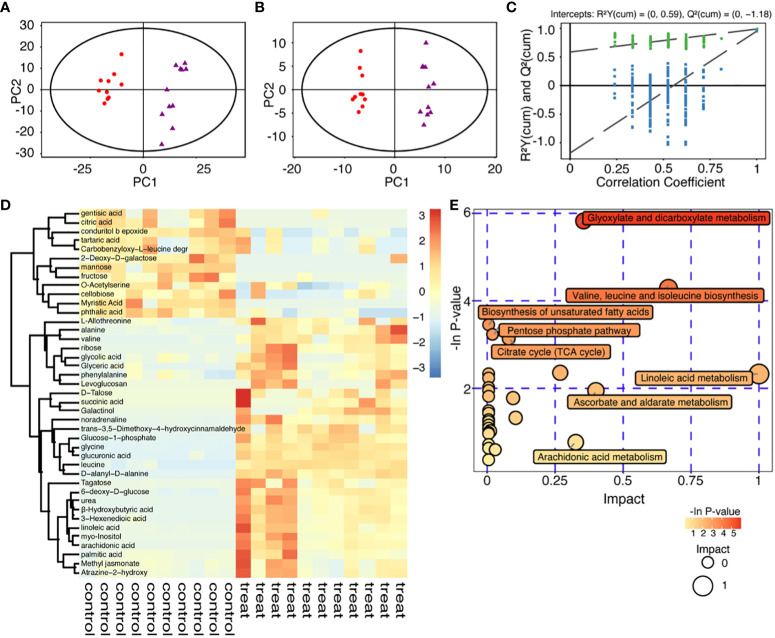
Metabolomic analysis of high glucose environment. **(A)** Principal component analysis (PCA) score plot of 40mM glucose groups and control groups. The X-axis and Y-axis were labeled with PC1 (the first principal component) and PC2 (the second principal component), respectively. 40mM glucose groups and control groups were scattered into two different regions. One data point stands for one sample: red dots, controls; purple triangles, treatments. **(B)** Orthogonal Partial Least Square-Discriminant Analysis (OPLS-DA) score plot from the 40mM glucose group and the control group, red dots represent controls, while purple triangles represent treatments. **(C)** Validation of the OPLS-DA model, green dot, R^2^; blue square, Q2. R^2 = ^0.988 and Q^2 = ^0.951, after 200 permutations. **(D)** Clustering heat map of significantly changed metabolites: Each sample was represented as a column (10 controls and 11 treats), and each metabolite was represented as a row. Metabolite abundance was represented by color: deep red, highest; deep blue, lowest; white, 0. **(E)** Functional enrichment (p-values) and pathway topology analysis (pathway impact) correlated with embryo development inhibited by high glucose. The x-axis represented the pathway impact, and the y-axis represented the pathway enrichment. Larger sizes and darker colors respectively represented the pathway enrichment and high pathway impact values.

**Figure 4:**
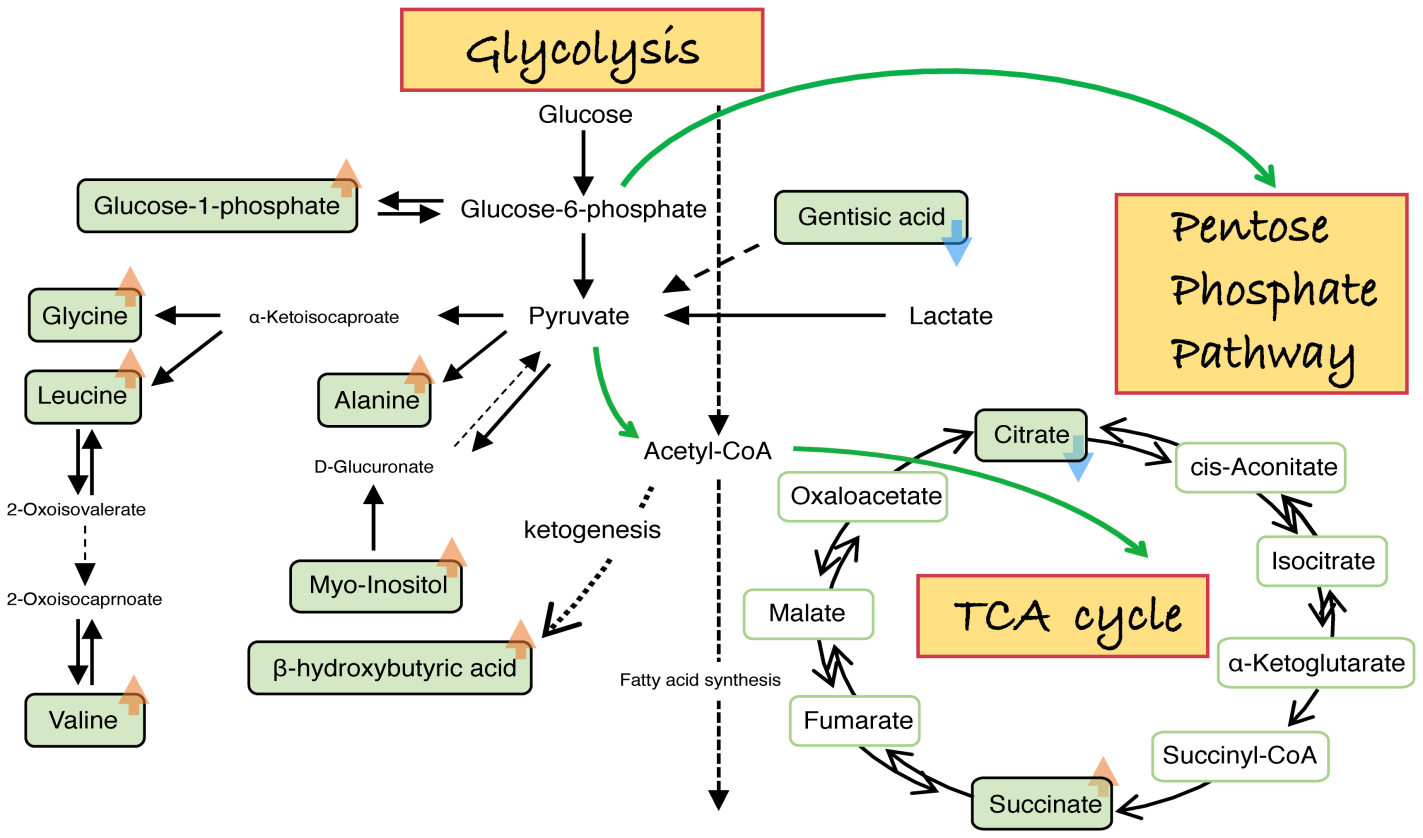
Schematic diagram of aberrant amino acids related to embryo development inhibited by high glucose. Summary of metabolic pathways and targets affected by high glucose during embryonic development. Embryos stunted by high glucose inhibition release abnormally metabolites, mainly involving PPP, glycolysis and the TCA cycle. Orange arrow (“↑”) represents an increased value of the embryonic metabolite released to the culture medium in the treatments compared with the controls. Blue arrow (“↓”) represents a decreased value of the embryonic metabolite released to the culture medium in the treatments compared with the controls.

The principal component analysis (PCA) showed the distribution of original data (**Figure 3A**). Supervised orthogonal projections to latent structures-discriminate analysis (OPLS-DA) were applied to visualize the similarities and differences among the data sets of the control groups and 40mM glucose groups (**Figure 3B**). Besides, the OPLS-DA model was validated by performing permutation tests (n=200) to verify its reliability and validity, with R^2 = ^0.988 and Q^2 = ^0.951 indicating good prediction. All the R^2^ (green dots) and Q^2^ (blue boxes) values from the permuted analysis (bottom left) were significantly lower than the original values (top right), showing a low risk of over-fitting (**Figure 3C**). 41 known differential metabolites were identified (VIP>1 and P-value<0.05) and illustrated by a heat map (**Figure 3D**). They were strongly linked to the inhibition resulting from a high glucose environment in embryonic development. These metabolites were mainly involved in glyoxylate and dicarboxylate metabolism; valine, leucine and isoleucine biosynthesis; biosynthesis of unsaturated fatty acids; pentose phosphate pathway; Krebs cycle (TCA cycle); linoleic acid metabolism; ascorbate and aldarate metabolism and arachidonic acid metabolism. Altered metabolites related to the inhibition of embryonic development by high glucose are mostly involved in energy metabolism and amino acid metabolism (**Figure 3E**). Specifically, embryonic development arrest due to high glucose was related to the aberrant amino acids released into the culture medium (**Figure 4**).

## Discussion

### Excess glucose originates from the diabetic mother

We support the view that the excess glucose in the embryonic environment originates from the diabetic mother. Though not directly relying on blood circulation, embryonic metabolism is closely connected to the maternal condition. The source of glucose in follicular fluid is plasma (58, 59) and metabolites found in the blood of a diabetic mother are transferred to the follicular fluid through the vascular theca. And the concentration of glucose in mouse fallopian tube fluid is similar to that in serum glucose (60). In addition, the metabolites in the blastocoel fluid correlate closely with that in the maternal blood (61, 62). In bovine follicular fluid, glucose concentration is significantly higher than that in serum (59). According to numerous studies on mice, we believe that 25-50 mM glucose is a reasonable concentration for preimplantation embryo development arrest due to high glucose. For women with diabetes, it is not only the absolute high blood glucose value. Even a slight increase in the fluctuation range increases the risk of abnormal embryonic development. Thus, we emphasize the importance of tight glycemic control in diabetic women at the earliest stages after conception. Attention needs to be paid to the condition of Impaired glucose tolerance (IGT), two-hour glucose levels of 7.8 to 11.0 mmol/L on the 75-g oral glucose tolerance test, whether the fasting glucose may be normal or mildly elevated.

### Embryonic diapause and glucose level

The existing literature generally believes that 15mM-28mM is a reasonable concentration for embryo diapause caused by diabetes embryopathy. In our study, five concentrations of glucose were set (control, 20mM, 40mM, 60mM and 80mM). The total cell number in 20mM glucose decreased, but the blastocyst rate showed no significant difference compared to the control group (**Figure 1D**). And the blastocyst rate was significantly decreased in 40mM, 60mM and 80mM glucose. Moreover, the blastocysts in 60mM glucose were poorly developed with low total cell number (**Figure 1E**), and no blastocysts were found in the 80mM glucose groups (**Figure 1D**). Therefore, 40 mM glucose is suitable and valid in our culture system. It arrested early embryonic development, for both the blastocyst rate and total cell number of the blastocyst decreased significantly. Interestingly, our results revealed that 60mM glucose and 80mM glucose content in the embryo culture had no effect on the 2-cell morphology and the cleavage rate (**Figure 3**), which echoed this literature (63). Similarly (11), high glucose content inhibited blastocyst development, and a significant decrease in blastocyst rate was observed as the glucose concentration was increased. In addition, a dose-related decrease in total and trophectoderm cell counts was associated with glucose, which was consistent with previous studies (11, 64). Like a previous report (52mM glucose) (10, 32), 40mM glucose was effective in inhibiting embryonic development in our study. Presumably, the slight difference in results was related to mouse strains, culture system and experimental procedures. The ability to develop to the blastocyst stage was identified as an indicator of embryo potential (65). And the inner cell mass morphology and cell number also appear to be effective parameters (66). This culture system successfully demonstrated the inhibitive properties of high glucose content as both the blastocyst rate and blastocyst total cell number were decreased correspondingly.

### Diapause of embryos under high glucose conditions and its relation to glucose transporters

In mouse embryo observations, a diet of 5015 resulted in elevated Maternal hyperglycemia potentially exacerbating the lack of coordination between genetically delayed neural folding and the fundamental tissues of normal development, thereby increasing the risk of neural tube malformations (67). In addition, the study on the mechanism of embryonic diapause in wild animals such as bats revealed that the enhanced glucose absorption by adipose tissue during adipogenesis was indicated by increased expression of insulin receptor (IR) and glucose transporter protein (GLUT4) proteins. The reduction in glucose uptake was associated with a decrease in insulin receptor (IR) and GLUT4 protein expression (68). Comparable experiments have also noted a reduction in the expression of GLUT3 and GLUT4 in gastrula-stage embryos of wild bats experiencing delayed development (69). Furthermore, in the high glucose model of chicken embryo diapause, chick embryos increased the average blood glucose concentration from 70mg/dL to 180mg/dL. They found a decrease in gene expression of glucose transporter GLUT1 (70).

Our experimental findings suggested that the high glucose group exhibited a rise in apoptosis and a decline in the proliferation of blastocyst cells (**Figure 2**). The inhibition of embryonic development by high glucose is linked to a decrease in the expression of glucose transporters (GLUT1, GLUT2, GLUT3). Earlier studies indicated a reduction in both transcripts and protein synthesis of the glucose transporters GLUT1 and GLUT3 in blastocysts cultured with either 25mM or 55mM glucose (71). In the blastocyst of a diabetes animal model, a 90 ± 5% decrease in GLUT2 protein and an 84 ± 6% decrease in GLUT3 protein were observed (35), along with a 63% decrease in GLUT2 mRNA and a 77% decrease in GLUT3 mRNA levels (72). Thus, studies have conclusively shown that embryo arrest related to maternal diabetes is marked by a decrease in the expression of glucose transporters. However, there is limited evidence indicating that the combination of embryonic stagnation and environmental changes may provide valuable insights. We posit that metabolites produced during embryonic development can reflect their current state characteristics. We investigated the metabolic characteristics of the embryonic development environment.

### The appropriate ratio, or “switch” of glycolysis and oxidative phosphorylation was disrupted by excess glucose

It has been well documented that the pentose phosphate pathway (PPP) plays a positive role in cells undergoing a large increase in biomass like proliferating cells and stem cells. Prenatal growth retardation, hydrops fetalis, dysmorphic features and congenital heart defects are strongly associated with PPP deficiencies (73). PPP is a branch of glycolysis. Although we did not directly detect perturbations of PPP in the embryonic developmental environment in our study. Based on the famous Crabtree effect and Warburg effect, it is natural for us to propose such a conjecture. The appropriate ratio or rate of glycolysis and oxidative phosphorylation was disrupted by excess glucose. The glucose-6-phosphate released by embryos in the high glucose group showed no significant difference from the control group (**Figure 4**). However, blastocysts with high viability demonstrated faster cleavage, larger inner cell mass and a lower glycolysis rate. In contrast, low viability blastocysts indicated the opposite (74, 75). In addition, the glycolytic rate was expressed as the conversion rate of glucose to lactate (76–78). As illustrated in **Figure 4**, t the lactate and pyruvate (glycolysis process) released by embryos in the high glucose group were not significantly elevated, with no significant difference from the control. This indicated that the glycolytic rate in the high glucose group was not elevated, which is in accordance with a previous study (79).

Blastocysts experience a lower oxygen concentration compared to 2-cell embryos (80). Both glycolysis and oxidative phosphorylation are active in the blastocyst stage, while the 2-cell embryo only relies on oxidative phosphorylation as energy metabolism (81). For cells undergoing a large increase in biomass like embryonic cells and cancer cells, glycolysis is a unique metabolic requirement. However, glycolytic conversion is an inefficient way of producing ATP. On the one hand, glycolysis might be related to reactive oxygen species, as efficient ATP production leads to reactive oxygen species (ROS) accumulation. At the embryonic stage, reactive oxygen species accumulation may cause fatal damage to the embryo. Thus, concurrent oxidative phosphorylation and glycolysis provided a suitable alternative to relying solely on oxidative phosphorylation. This energy-generating pattern with a high glycolytic rate could contribute to diminishing oxidative stress and simultaneously avoid ROS generation (82). On the other hand, embryos acquire and metabolize nutrients to promote proliferation rather than efficient ATP production, which is the same as cancer cells (83). Glycolysis provides the fastest response to meet the proliferation requirements, although not being the most effective way to produce energy. Notably, glycolysis produces ATP faster in the presence of excess glucose and oxygen deficiency, which is common among rapidly proliferating cells such as embryonic cells and cancer cells (81, 83). The significance of glycolysis is a unique metabolic requirement rather than an energy requirement.

### Decreased citrate and increased succinate levels implicating impaired mitochondrial metabolism

Our results showed decreased citrate and increased succinate levels in the high glucose culture medium. It raised the intriguing possibility of a deranged TCA cycle under high glucose conditions. As citrate regulates the balance between glycolysis and oxidative phosphorylation (76, 77, 84), a loss of such a balance may impede the proliferation of embryonic cells. Embryos acquire and metabolize nutrients for proliferation rather than efficient ATP production, which is the same in cancer cells (83). Moreover, disordered glycolysis, Krebs cycle, and oxidative phosphorylation may lead to poor mitochondrial activity and inefficient adenosine triphosphate (ATP) production. Meeting the immediate need for ATP is prioritized over generating the maximum amount of ATP. Mitochondria’s ability to balance ATP supply and demand is the key to embryonic development (78).

The metabolic changes in early embryo apoptosis induced by high glucose suggested an imbalance in energy management. The inability of mitochondria to balance ATP supply and demand may result from an instantaneous ATP flux disorder. The energy production, such as the normal ratio of glycolysis, Krebs cycle and oxidative phosphorylation, might be disrupted. As opposed to glycolysis, oxidative phosphorylation is present in embryos at all stages of preimplantation development. Energy production is important to cells undergoing a large increase in biomass like embryonic cells and cancer cells. Notably, a similar experiment was performed in a previous study using a glucose concentration of 50mM. However, only the blastocysts were measured, not the culture medium. Blastocysts exposed to high glucose concentrations (50 mM) demonstrate an increase in TCA cycle metabolites, increased pyruvate, and a decrease in glycolytic metabolite production (79). Another study indicated high levels of Krebs cycle metabolites such as citrate and fructose from low-quality blastocysts cultured in 52mM glucose (85). The above findings supported our conclusions.

### Aberrant amino acid metabolism in glycolysis

The equilibration of metabolite concentration between embryos and the culture medium is relatively slow (86). Therefore, measuring its turnover using only amino acid appearance or depletion in the culture environment is inaccurate (54, 87). However, information can still be obtained via the amino acids released by those embryos in the culture media, although measuring amino acid turnover is complex.

Glycine and alanine are highly abundant amino acids in oviductal fluid and follicular fluid and are crucial for preimplantation development. They regulate cell osmotic pressure, maintain cell volume, and prevent ion concentration from rising to a level inhibiting embryo development (87, 88). Furthermore, they contribute to withstanding external high osmolarity conditions (89–91)and enhance embryonic development (92). Increased glycine and alanine have been associated with embryo arrest prior to the blastocyst stage (54). Interestingly, embryos arrested in the same stage in high glucose conditions released more glycine and alanine into the culture media than the normal ones. The results suggest that poor-quality embryos exhibit a lower rate of glycine uptake (87). Glycine transport might be impaired due to long-term exposure to a high glucose environment. It is possible that those embryos that failed to develop were already deficient in glycine uptake. The discrepancy between our observations and other reports (52, 53) may be attributed to the difference in experimental methods, such as different embryonic stages, animal models, culture systems, and culture volume (20 embryos per 75μL). In addition, mouse embryos in the present study were naturally fertilized.

Moreover, increased myo-inositol levels were also observed in the high glucose culture medium. This may potentially be caused by a decreased uptake of myo-inositol, with a slow equilibration between the embryo and the culture medium. Weigensberg et al. reached similar conclusions (86). High glucose-induced teratogenesis was mediated by the myo-inositol deficiency of embryos. A myo-inositol depletion study in high glucose-induced abnormal embryonic development showed a significant decrease in myo-inositol content of the 9.5-day embryo compared to the control group after the addition of 33.3 mmol/l and 66.7 mmol/l glucose to the culture media (93). In addition, Weigensberg et al. characterized the uptake of myo-inositol by 10.5-day rat conceptus. The increased ambient glucose competitively inhibited net myo-inositol uptake in a concentration-dependent fashion (86) which is consistent with our expectations.

The embryonic development inhibition by high glucose was also manifested by an increase in leucine, valine and β-hydroxybutyric acid (β-OHB) in the culture medium. The results are consistent with the animal research of McKiernan et al. (94). In addition, higher levels of leucine and valine were observed in the plasma of human diabetic mothers (95, 96). Notably, metabolites in the blood of a diabetic mother can be reflected in her follicular fluid and blastocoel fluid (61, 62). β-OHB is significantly higher in the first trimester of diabetic mothers (97). Animal research has demonstrated that β-hydroxybutyric acid (β-OHB) causes developmental toxicity in embryonic development (98, 99) with dose-dependent effects (100). In conclusion, β-OHB decreases viability, cleavage rates and blastocyst formation rates (**Figure 4**) (57).

### The osmolarity of the culture medium

First, the culture system used in this study contained glutamine. Glycine is produced by embryo metabolism. Osmolarity imbalances were unlikely due to the presence of these amino acids in the culture medium (88, 91). Second, no significant changes in embryo volume were found in the high glucose group compared with normal embryos. When exposed to an external hypertonic medium, solutes are actively transported against a concentration gradient into the embryo to reduce the activity gradient (active transport), preventing water from flowing out of the cell down the water activity gradient (passive transport) (101). Third, osmolarity imbalances lead to the zygote’s arrest at the 2-cell stage (63, 101). However, our results showed that early embryos were not inhibited. Furthermore, Wyman et al. used a 52 mM D-glucose culture system to mimic maternal diabetic conditions as they consider it most closely replicates the diabetic blastocyst phenotype (10, 32). We believe the osmolarity in our culture system was suitable since the zygote development was not adversely affected by the 40mM glucose medium. Moreover, no additional sodium hydroxide solution was used to adjust the osmotic pressure considering that a certain degree of osmotic pressure might be caused by a maternal diabetic condition.

### High lights

The *in vitro* mouse embryo model was meaningful and reliable not only because it was sensitive to changes in nutrient supply but also because it closely resembled that of human preimplantation embryos. Elevated glucose levels hinder the early development of embryos. As the glucose concentration increased, the inhibitory effect notably intensified within the 20mM-80mM concentration range. Surprisingly, the 2-cell morphology and cleavage rate are unaffected by glucose levels of 80mM glucose in embryo culture. We analyze the metabolites in the culture medium of diapause embryos, a study that has not been reported previously. Cause we posit that the metabolites produced during embryonic development can reflect the current state characteristics of the embryos. Additionally, collecting a substantial number of embryos suppressed by high sugar and extracting sufficient proteins poses challenges. While there are previous reports on immunofluorescence results, there is a scarcity of information on immunoblotting results. In our study, we have completed this task and we are willing to share our embryo culture system with interested scholars. Details are provided in the supplementary documents.

## Data availability statement

The raw data supporting the conclusions of this article will be made available by the authors, without undue reservation.

## Ethics statement

The animal study was approved by the Beijing University of Chinese Medicine Animal Ethics Committee. The study was conducted in accordance with the local legislation and institutional requirements.

## Author contributions

JH (first author): Writing-First Draft, Embryo culture. GJ: experimental guidance, figure-making guidance. XW: resources, supervision. YL: statistics, visualization. XL: software, validation. LH: Review and editing. XY: Literature search. YW: Supervision, metabolism analysis guidance. KX: Supervision. QL Supervision, Consulting. QF: Resources, Methodology. TN (corresponding author): Formal Analysis, Methodology. XN (corresponding author): Conceptualization, Methodology. All authors contributed equally to this work and approved the submitted version.

## Supplementary material

Supplementary Table 1 The formula of M2 Operating Fluid.

Supplementary Table 2 The formula of Culture Media.
